# Perioperative Management of a Patient With von Recklinghausen’s Disease With Anticipated Difficult Airway Management: A Case Report

**DOI:** 10.7759/cureus.22713

**Published:** 2022-02-28

**Authors:** Aiji Sato-Boku, Izumi Kuroda, Hideto Imura, Mayumi Hashimoto, Naoko Tachi, Masahiro Okuda

**Affiliations:** 1 Department of Anesthesiology, Aichi Gakuin University, Nagoya, JPN; 2 Cleft Lip and Palate Center, Aichi Gakuin University, Nagoya, JPN

**Keywords:** mandibular osteomyelitis, multiple neurofibromatosis, airway management, awake intubation, von recklinghausen's disease

## Abstract

Von Recklinghausen's disease is characterized by skin pigmentation, multiple neurofibromatosis, and osseous changes. In the anesthetic management of patients with von Willebrand's disease, it is important to provide appropriate airway management, taking into account the cutaneous laxity caused by neurofibromatosis of Recklinghausen's disease. This case describes the perioperative management of a patient with Recklinghausen's disease with suspected difficulty in airway management.

## Introduction

Von Recklinghausen's disease, which was first reported by Friedrich Daniel von Recklinghausen in 1882, is characterized by skin pigmentation, multiple neurofibromatoses, and osseous changes [1]. The disease occurs in one out of 3000-4000 individuals. Problems in anesthesia management include hypertension caused by renal artery stenosis and circulation instability and major bleeding caused by pheochromocytoma; airway obstruction caused by a tumor in the oropharynx and larynx; and an aberrant response to muscle relaxants [2-5]. Here, we report the perioperative management of a patient with suspected difficulty in airway management because of cutaneous laxity caused by neurofibromatosis of Recklinghausen's disease and trismus resulting from pathological fractures associated with mandibular osteomyelitis. The patient provided written informed consent.

## Case presentation

**Figure 1:**
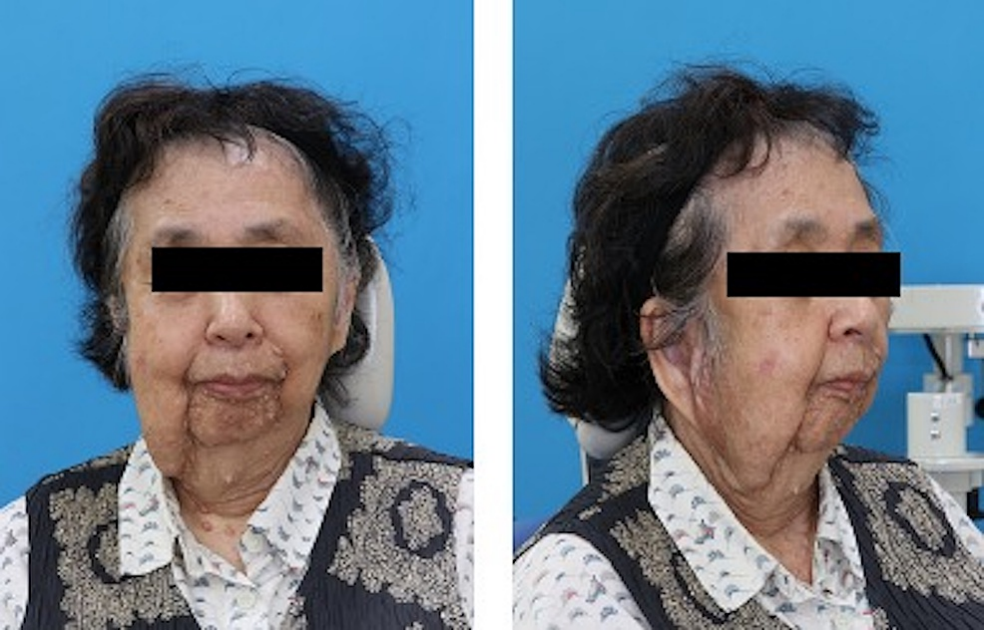
Front and side view of the patient Cutaneous laxity (left) and mandibular micrognathia (right) caused by neurofibromatosis of von Recklinghausen's disease

**Figure 2:**
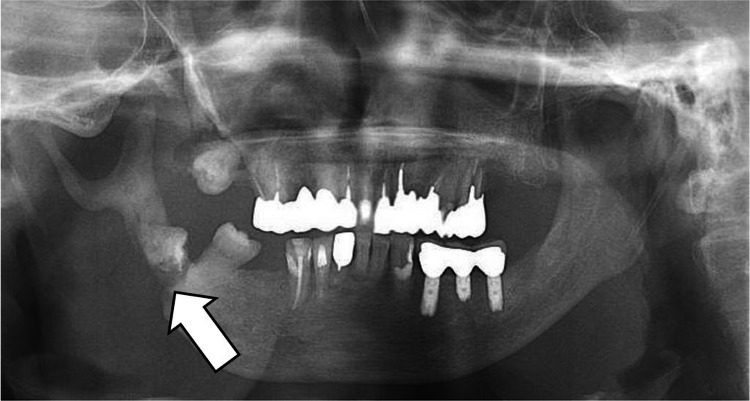
Panoramic radiograph of the patient Pathological fractures associated with mandibular osteomyelitis

A 74-year-old woman (height, 151 cm; weight, 58 kg; body mass index (BMI) 25.7) was scheduled for tooth extraction and anti-inflammatory surgery under general anesthesia for right mandibular osteomyelitis. She had been diagnosed with von Recklinghausen's disease at an early age; her mother also had the same disease. Her past medical history included surgery for appendicitis when she was 50 years of age. Her preoperative examination findings, including blood tests, chest radiography, electrocardiogram, and vital signs, were uneventful, except for mild hypertension noted by an internist. In addition, airway management was predicted to be difficult based on the following preoperative airway findings: cutaneous laxity (Figure 1, left) and mandibular micrognathia (Figure 1, right) caused by neurofibromatosis of von Recklinghausen's disease and trismus resulting from pathological fractures associated with mandibular osteomyelitis (Figure 2). There was no problem with the range of motion of the cervical spine. The Maranpathi classification was 3 and the inter-incisor distance was two lateral fingers.

Considering the airway findings, mask-to-face ventilation after anesthesia induction was deemed impossible; therefore, we chose awake fiberoptic intubation while maintaining spontaneous ventilation under sedation. When the patient entered the operating room, standard monitors were attached. No major problems were noted with her vital signs, except for a systolic blood pressure of between 180 and 190. After establishing an intravenous 20-gauge line in the left forearm and adequate oxygenation at 6 L/min via face mask, we administered 2 mg of midazolam and 50 µg of fentanyl. Once sedated, her systolic blood pressure decreased to 120-130. After confirming sufficient sedation and spontaneous breathing, we performed cricothyroid cartilage puncture under ultrasound guidance using 2 ml of 4% xylocaine. After doing cricothyroid cartilage puncture, cough reflex was observed. Appropriate nasal preparation was performed and awake intubation was performed using a fiberscope from the right nasal cavity. The nasotracheal tube used in this case was Polar^TM^ Preformed Tracheal Tube (Smith Medical Japan Ltd, Tokyo), and the tube size was ID 6.5 mm. Airway findings through the fiberscope were unremarkable, with no narrowing due to neurofibromatosis. The intubation was easy and took about one to two minutes. Thereafter, anesthesia was induced with 50 mg of propofol and 40 mg of rocuronium. Intraoperatively, anesthesia was maintained using oxygen, air, desflurane, fentanyl, and remifentanil. Postoperatively, the patient recovered from the muscle relaxants, as confirmed by neuromuscular monitoring. Then, 150 mg of sugammadex, a rocuronium antagonist, was administered. After body movement, eye-opening, adequate spontaneous breathing, and obedience were confirmed, she was extubated using a tube exchanger. After observing the patient for a while and confirming that there was no problem with her respiratory condition, the tube exchanger was removed. Subsequently, the systolic blood pressure increased to 180-190. Other vital signs remained within the normal range. The operation lasted for one hour and four minutes, with two hours and 12 minutes of anesthesia. The amount of blood loss was 30 g.

## Discussion

As described above, various problems are associated with anesthesia management in patients with von Recklinghausen's disease. In the present case, the worst problem in anesthesia management was the difficulty in establishing an intact airway. Generally, mask-to-face ventilation is difficult or impossible in patients with upper-airway deformity because of several factors, including tumor, abscess, radiation history, and surgery history [6-7]; thus, awake intubation is often indicated. Although awake intubation is safe because it is performed while maintaining the patient's spontaneous breathing, it must be chosen after careful consideration because it can be painful if the anesthesiologist is not skilled. The procedure cannot be performed without patient cooperation. In addition, persistent airway injuries have been reported [8]. In our case, mask-to-face ventilation was also impossible because of cutaneous laxity caused by neurofibromatosis, which is a characteristic of von Recklinghausen's disease. Therefore, we had to choose awake intubation. The use of midazolam, fentanyl, and a local anesthetic agent to reduce the patient's pain allowed uneventful awake intubation. One preventive measure against respiratory depression is the insertion of a tracheal tube through the nasal cavity. The tube’s tip is placed close to the glottis, serving as an airway if oxygenation is reduced due to insufficient spontaneous ventilation during awake intubation under sedation. Fortunately, such a tube was not needed in the present case. A sedative agent with minimal respiratory depression, such as dexmedetomidine, maybe a better choice when the impossibility of mask-to-face ventilation is suspected.

Another problem we encountered during anesthesia management was the increase in systolic blood pressure, which is similar to a previous report [2]. Previous reports have documented cases of massive bleeding, which is a characteristic of von Willebrand disease, in addition to difficulty in intraoperative blood pressure management [2]. In our case, the systolic blood pressure was elevated to 180-190 owing to tension upon entering the operating room and stimulation during extubation. Thus, the possibility that the patient had hypertension concomitantly cannot be ruled out. The patient’s blood pressure was slightly high before the operation, which was noted by an internist. Therefore, preoperative interventions, such as the administration of antihypertensive medication, should have been performed. Although no major circulatory changes were noted intraoperatively, adequate consideration of the circulatory system may be necessary even if the patient did not report any problems.

Furthermore, the effects of muscle relaxants persist in some cases [3-4]. In our case, the muscle relaxant effect subsided although it was monitored continuously by neuromuscular monitoring during the operation. To ensure safety, we reversed the muscle relaxant before awakening the patient from anesthesia. In the case we experienced, there was no residual muscle relaxant but since the tracheal intubation was performed under conscious intubation without muscle relaxants, it may not have been necessary to use muscle relaxants intraoperatively and risk their prolongation.

## Conclusions

We experienced the perioperative management of a patient with suspected difficulty in airway management because of cutaneous laxity caused by neurofibromatosis of Recklinghausen's disease and trismus resulting from pathological fractures associated with mandibular osteomyelitis. Although we were able to secure the airway by conscious intubation without any problems, there were some points to be considered regarding intercirculatory management and the use of muscle relaxants.
